# Supplementary material for: Strong in-plane scattering of acoustic graphene plasmons by surface atomic steps
Source: Nat Commun. 2022 Feb 21;13:983. doi: 10.1038/s41467-022-28614-z (PMC8861092; doi:10.1038/s41467-022-28614-z)
Supplement: Supplementary file 1 — Supplementary Information [file 41467_2022_28614_MOESM1_ESM.pdf]

Supplementary Information: Strong in-plane scattering of acoustic graphene plasmons by surface atomic steps

Ni Zhang,[1,*] Weiwei Luo,[1,*] Lei Wang,[2,*] Jiang Fan,[1] Wei Wu,[1]
Mengxin Ren,[1] Xinzheng Zhang,[1] Wei Cai,[1,3,†] and Jingjun Xu[1,‡]

[1] *The Key Laboratory of Weak-Light Nonlinear Photonics, Ministry of Education, School of Physics and TEDA Institute of Applied Physics, Nankai University, Tianjin 300457, China*

[2] *College of Physics and Electronic Engineering, Xinyang Normal University, Xinyang 464000, China*

[3] *Collaborative Innovation Center of Extreme Optics, Shanxi University, Taiyuan, Shanxi 030006, People's Republic of China*

(Dated: January 10, 2022)

[*] Contributed equally to this work
[†] Electronic address: weicai@nankai.edu.cn
[‡] Electronic address: jjxu@nankai.edu.cn

Supplementary Note 1: Sample preparation

Monolayer graphene and thin layer h-BN were prepared by mechanical exfoliation from flaky graphite and hexagonal boron nitride crystal using the method in Ref. [1], which were then transferred onto resistive silicon wafers with 300 nm of thermally-grown oxide. The thickness of the graphene and h-BN is characterized by the atomic force microscope (AFM).

The hBN/graphene/hBN heterostructure devices were assembled by the polymer-free van der Waals assembly technique [2]. The transfer process is illustrated step by step in Supplementary Figure 1. Firstly, through changing the temperature, the top h-BN, graphene and bottom h-BN can be repeatedly picked up by the PC film and form heterostructure due to the viscosity control. Finally, the whole heterostructure is placed on the target substrate and PC is removed by rinsing in chloroform for 45 s. The Au electrodes in the target substrate were obtained using standard ultraviolet lithography and electron beam evaporation techniques.

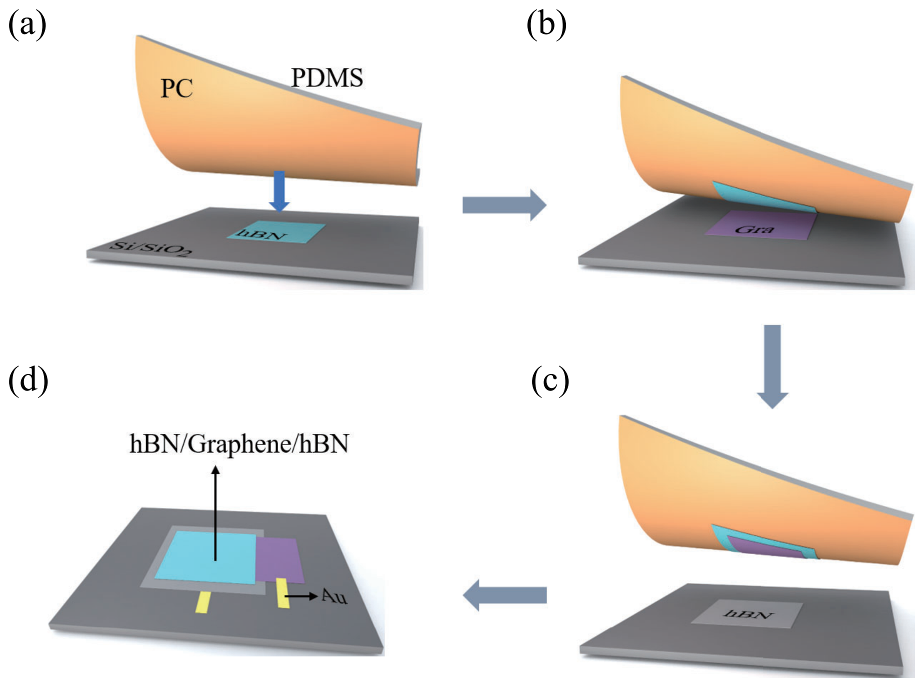

Supplementary Figure 1. The fabrication process of the heterostructure devices. (a) The PC film catches the top h-BN. (b, c) The top h-BN catches the monolayer graphene and bottom layer h-BN. (d) The heterostructure is placed on the target substrate.

Supplementary Figure 2 shows the optical microscopic image of the devices in the main article, the contacts are formed with Au electrodes (50 nm in height). The encapsulated

region consists of top h-BN, monolayer graphene and bottom h-BN, which are shown as the red, black and white lines, respectively. The bottom h-BN is used as a spacer between graphene and Au.

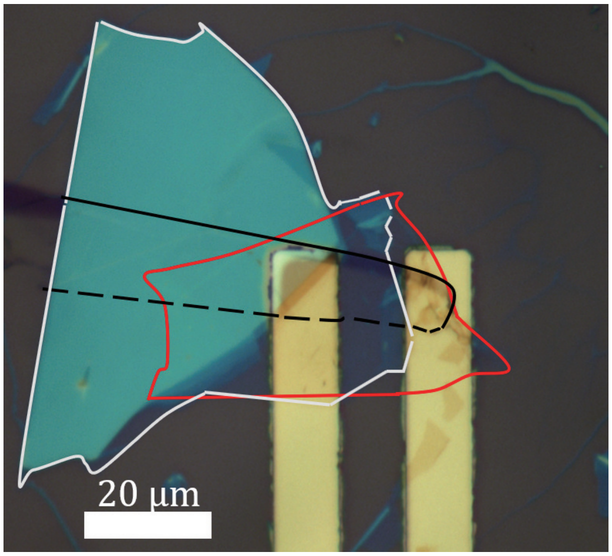

Supplementary Figure 2. The image of the prepared device under optical microscopy. The red, black, and white lines enclosed areas represent the top h-BN, the middle layer graphene and the bottom h-BN, respectively. Scale bar, 20 μm

Supplementary Note 2: The unflatness of the Au substrate

The AFM was used to analyze the morphology of the Au substrate before and after the transferring of the hBN/graphene/hBN heterostructure on it, which shows that the roughness is about 6 nm and 3 nm, respectively. As a result, we can estimate the encapsulated region is partially suspended on the holes. The depth of the holes is around 3 nm, which is given by the depth difference between bare Au substrate and our text sample. The AFM topography of the bare Au substrate and the encapsulated region on top of Au substrate is shown in Supplementary Figure 3.

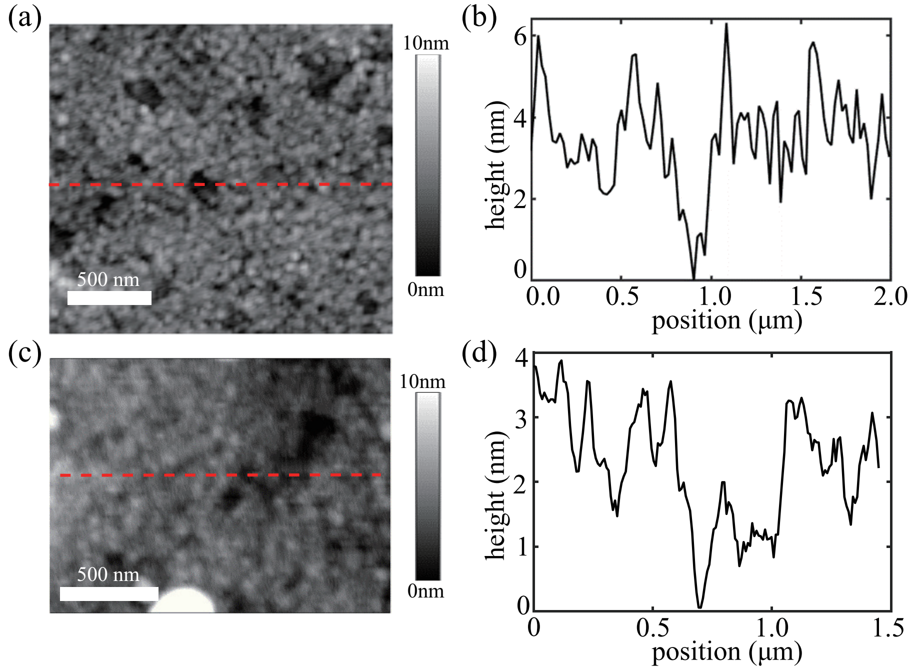

Supplementary Figure 3. Comparison of AFM topography of the bare Au substrate and the heterostructure stacked on top of the Au substrate. (a) AFM topography of the bare Au substrate. (b) The line profile of height corresponds to the dashed red line in (a). (c) AFM topography of the encapsulated sample region. (d) The line profile of height corresponds to the dashed red line in (c). Scale bars, 500 nm

Supplementary Note 3: The comparison between the AFM topography and the corresponding near-field image of the scatterers

To confirm that the plasmon standing waves come from the plasmon scattering of nanometer steps, the AFM topography of sample 1 and corresponding near-fields for the same area are given in Supplementary Figure 4. From the wide field of view, the correlation between steps and near-field signal is not obvious. As a result, two separate regions labeled as I and II were chosen. The line distribution of the AFM and near-field data in these two regions are given in Supplementary Figure 4(c) and (d) and Supplementary Figure 4(e) and (f), respectively. One can find the near-fields signal appears probably due to unflatness of the surface, which exists in both directions away from the steps. However, the signal is not consistent with a typical reflection of plasmons by a boundary, which can be attributed to the collective multiple scattering due to the large density of the scatters.

As a result, the sample 2 with a low density of scatterers is examined. Two independent

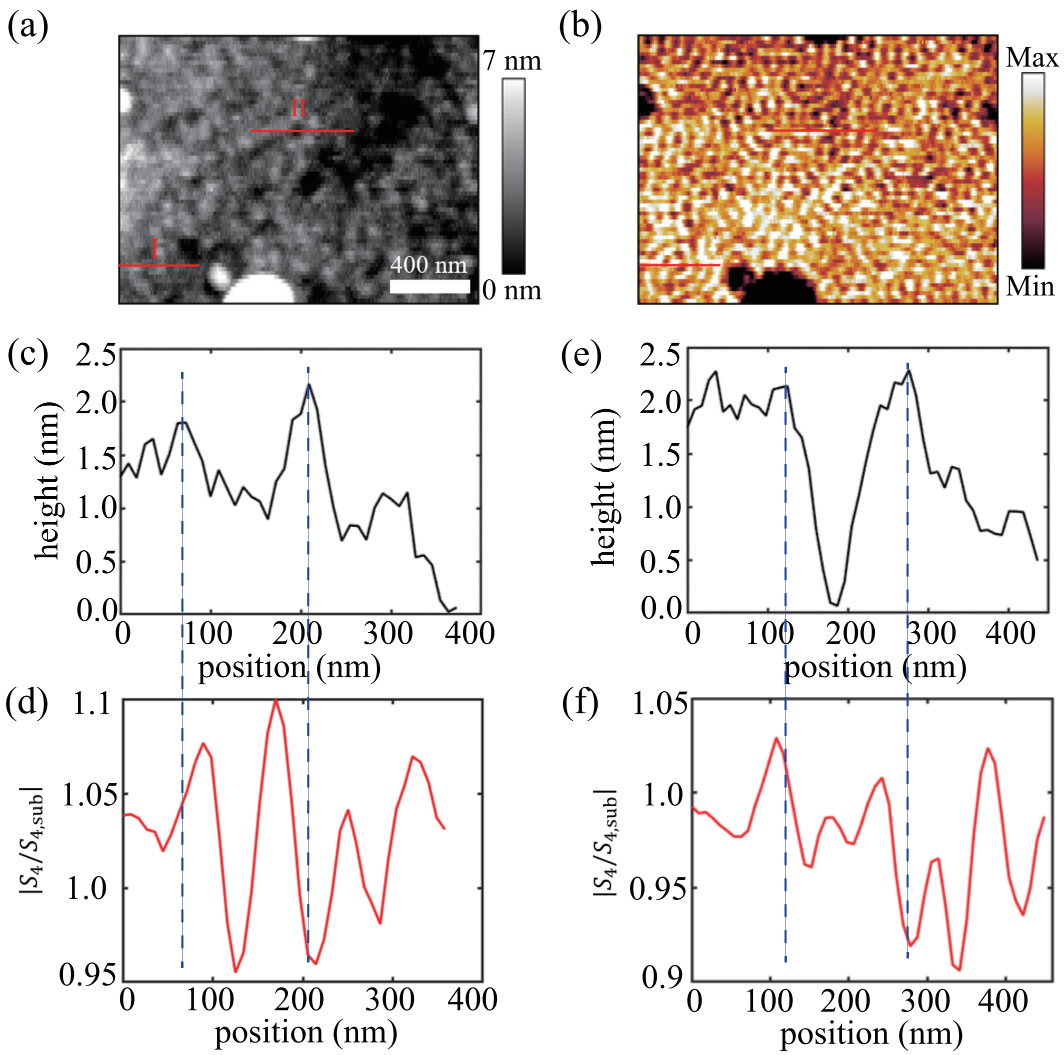

Supplementary Figure 4. The AFM topography and the corresponding near-field image of the Fig. 1 in the main article. (a) The AFM topography. (b) The near-field image of (a). (c), (d) and (e), (f) is the line profile of the AFM and the near-field profile of I and II.

scatterers were selected and shown in Supplementary Figure 5. Supplementary Figure 5(a) and (b) are the AFM and corresponding near-field of the independent scatterers I and II. From the AFM morphology we can clearly see the position of the scatterer, where a hole is formed. Supplementary Figure 5(c) and (d) and Supplementary Figure 5(e) and (f) show the line profile of the AFM and the near-field profile shown by the white dotted line in Supplementary Figure 5(a) and (b). It is obvious from the field distribution that the fringes are caused by the scattering of scatterers, where near-fields exist outside the hole. Except for that, inside the hole region, the plasmon resonance also exists, which contributes additional momentum components in the dispersion plots in Fig. 1c and f in the main article.

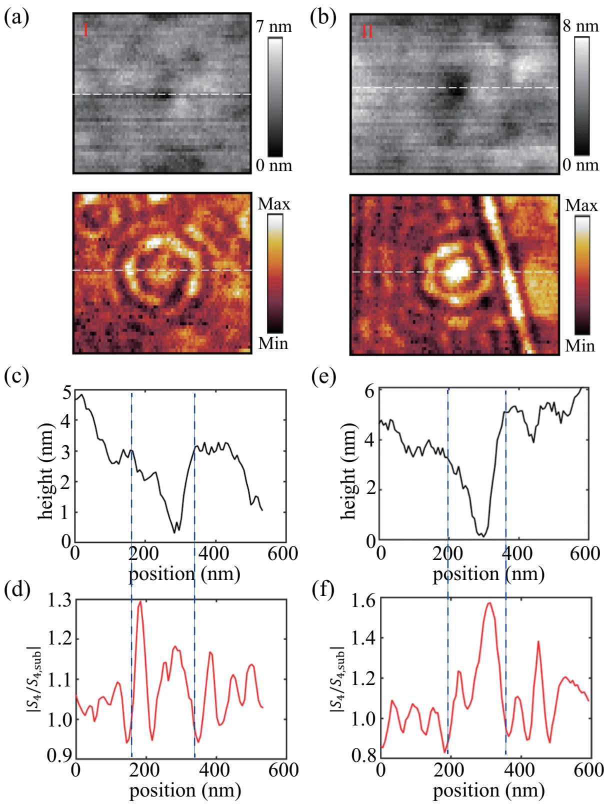

Supplementary Figure 5. The AFM topography and the corresponding near-field image of two independent scatterers. (a), (b) The AFM topography and the near-field image of the independent scatterers of I and II. (c), (d) and (e), (f) is the line profile of the AFM and the near-field profile shown by the white dotted line in I and II.

Supplementary Note 4: Material analysis of the plasmon scatterers

To determine which these plasmon scatterers presented in the substrate might stem from, Energy-dispersive X-ray Spectroscopy (EDS) analysis was adopted. When the acceleration voltage of the excited electrons is reduced to a certain range, the characteristic X-rays almost all come from the surface of the material, reflecting the composition information of the surface composition. Energy spectra of the SiO_2 and Au substrate before and after lithography under 4 kV acceleration voltage are shown as Supplementary Figure 6. In principle, the scatterers can be formed by nonuniform gold evaporation or possible chemical pollution during lift-off process. However, by comparing the characteristic peak intensities and atomic percentages of different elements (mainly C and O) before and after the lithography, one can find that there is no obvious increment for C and O and the possibility of photoresist

residues can be excluded. As a result, the scattering is coming from the unflatness of the Au film.

(a)

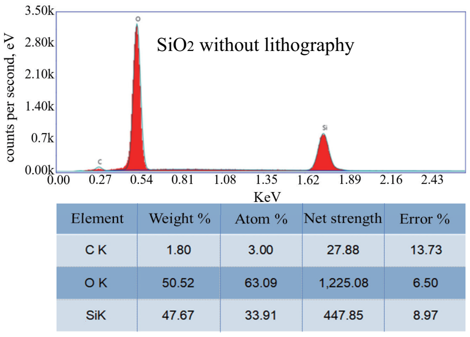

Element	Weight %	Atom %	Net strength	Error %
C K	1.80	3.00	27.88	13.73
O K	50.52	63.09	1,225.08	6.50
SiK	47.67	33.91	447.85	8.97

(b)

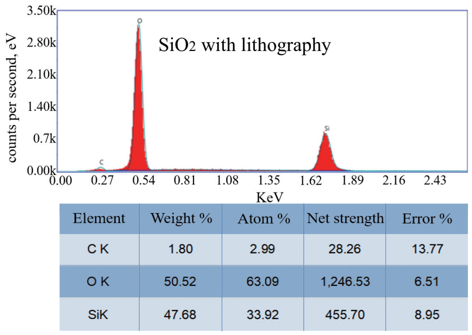

Element	Weight %	Atom %	Net strength	Error %
C K	1.80	2.99	28.26	13.77
O K	50.52	63.09	1,246.53	6.51
SiK	47.68	33.92	455.70	8.95

(c)

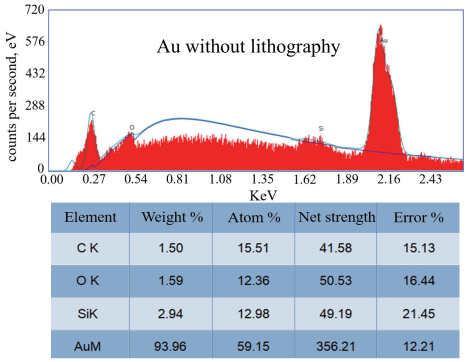

Element	Weight %	Atom %	Net strength	Error %
C K	1.50	15.51	41.58	15.13
O K	1.59	12.36	50.53	16.44
SiK	2.94	12.98	49.19	21.45
AuM	93.96	59.15	356.21	12.21

(d)

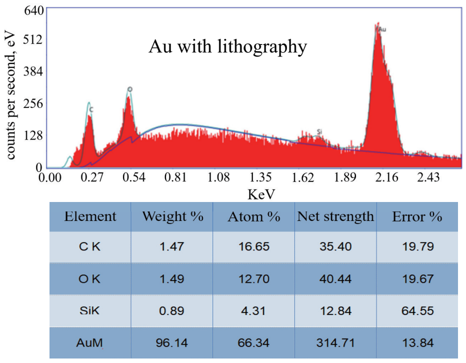

Element	Weight %	Atom %	Net strength	Error %
C K	1.47	16.65	35.40	19.79
O K	1.49	12.70	40.44	19.67
SiK	0.89	4.31	12.84	64.55
AuM	96.14	66.34	314.71	13.84

Supplementary Figure 6. The EDS spectra of the samples with different substrates. (a) SiO_2 substrate before lithography. (b) SiO_2 substrate after lithography. (c) Au substrate before lithography. (d) Au substrate after lithography.

Supplementary Note 5: Calculation of AGPs dispersion

In the calculation, the quantum capacitance effect needs to be considered for the thin hBN dielectric (6 nm). The relation between the carrier density n_s and the backgate voltage V_g can be written as [3, 4],

$$V_\mathrm{g} = \frac{V}{e} + en_\mathrm{s}/C_0, \tag{1}$$

where V is the internal chemical potential of the graphene, and C_0 is the areal geometric capacitance. The doping at zero gate voltage is very close to zero and can be ignored for the encapsulated sample.

Furthermore, to get a more accurate description of the conductivity of graphene within the acoustic plasmon response, wavevetor q dependence is included as [5],

$$\mathrm{Im}\{\sigma_\mathrm{graphene}(\omega, q)\} \approx \omega^{-1}e^2(2v_\mathrm{F}k_\mathrm{F}/h)[1 + \frac{3}{4}q^2v_\mathrm{F}^2/\omega^2], \tag{2}$$

where $k_{\rm F} = \sqrt{\pi n_{\rm s}}$ is the Fermi wavevector. The Fermi velocity renormalization effect [3, 4] can be ignored considering the high carrier density of the sample in our experiment.

Considering the above effects, the dispersion relation of acoustic graphene plasmons is obtained by using transfer matrix method. Firstly, considering the electromagnetic scattering at a single-layer graphene interface, in which the graphene sheet is sandwiched by two semi-infinite dielectric media characterized by the relative permittivities ϵ_1 and ϵ_2, as described in Supplementary Figure 7.

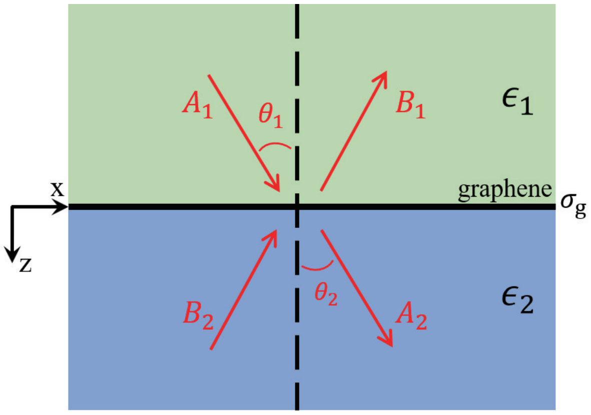

Supplementary Figure 7. Electromagnetic scattering at a single graphene 2D layer. The graphene sheet is located at the plane defined by $z = 0$, and cladded between two semi-infinite dielectric media characterized by the relative permittivities ϵ_1 and ϵ_2 as indicated in the figure. The electromagnetic properties of graphene are encompassed by its conductivity, $\sigma_{\rm g}$.

Therefore, the T-matrix across a single graphene [6], namely

$$T_{1\to 2} = \frac{1}{2}\begin{pmatrix} 1+\eta+\xi_\sigma & 1-\eta-\xi_\sigma \\ 1-\eta+\xi_\sigma & 1+\eta-\xi_\sigma \end{pmatrix} \tag{3}$$

And the T-matrix across an interface between two media in which the graphene sheet is absent, can be fetched from Supplementary Equation (3) by taking the limit when $\sigma_{\rm g}$=0, producing

$$T^0_{1\to 2} = \frac{1}{2}\begin{pmatrix} 1+\eta & 1-\eta \\ 1-\eta & 1+\eta \end{pmatrix} \tag{4}$$

For a multilayer system, the T-matrix can be written:

$$T_{1\to N} = T_{1\to 2}\cdot P_2(d)\cdots T_{M-1\to M}\cdot P_M\cdot T_{M\to N} \tag{5}$$

Where the "dot" denotes matrix multiplication, and P_M is a propagation matrix describing the free wave-propagation along a dielectric layer of length d and characterized by a relative permittivity ϵ_M, and it can be described as

$$P_M(d) = \begin{pmatrix} e^{-ik_{M,z}d} & 0 \\ 0 & e^{ik_{M,z}d} \end{pmatrix} \tag{6}$$

The matrix $T_{M-1\to M}$ is the T-matrix describing the propagation of light across a single graphene sheet. After the graphene layer, the electromagnetic radiation needs to propagating along an insulating slab of thickness d before reaching the next graphene layer; this is accounted for by the matrix $P_M(d)$. Finally, we just need to consider the effect of the next graphene layer by multiplying the previous matrices from the right with $T_{M\to N}$, which is the same T-matrix of single-layer graphene. The reflection and transmission coefficients of the multilayer system can be calculated from the elements of the transfer matrix as follows:

$$R_N = r_N^2 = \left| \frac{T_{1N}(2,1)}{T_{1N}(1,1)} \right|^2 \tag{7}$$

Using the above method, the reflection coefficient of our multilayer system shown as Supplementary Figure 8 has been simulated.

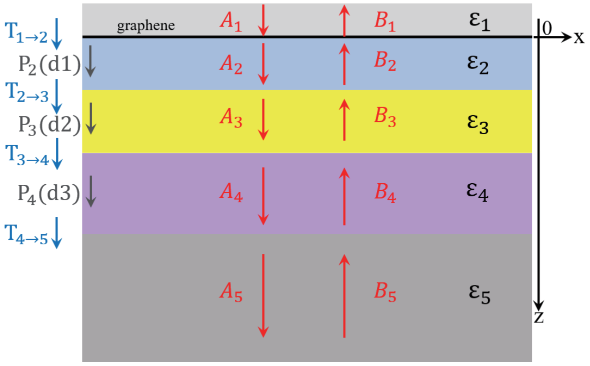

Supplementary Figure 8. Electromagnetic scattering of our devices (here depicted at normal incidence). The graphene sheet is located at $z = 0$ and are encapsulated by top h-BN (2 nm) and bottom h-BN (6 nm). The thickness of the Au and SiO_2 is 50 and 300 nm, respectively.

The multilayer system from top to down is h-BN (2 nm)/monolayer graphene/h-BN (6 nm)/Au (50 nm)/SiO_2 (300 nm)/Si. The h-BN is a dispersive material with a frequency dependent permittivity ϵ due to phonons. Due to the layered nature of h-BN, it is an anisotropic material and so its permittivity ϵ is a tensor. Choosing x, y to be the in-plane

Supplementary Table 1.h-BN permittivity parameters.

l	$\epsilon_l(\infty)$	$s_{v,l}$	$\hbar\omega_{v,l}$/meV	$\hbar\gamma_{v,l}$/meV
x, y	4.87	1.83	170.01	0.87
z	2.95	0.61	92.5	0.25

directions and z to be the out-of-plane direction, by symmetry the permittivity must be diagonal in a perfect h-BN crystal:

$$\epsilon = \begin{pmatrix} \epsilon_\mathrm{x} & 0 & 0 \\ 0 & \epsilon_\mathrm{y} & 0 \\ 0 & 0 & \epsilon_\mathrm{z} \end{pmatrix} \tag{8}$$

with components $\epsilon_\mathrm{x} = \epsilon_\mathrm{y} \neq \epsilon_\mathrm{z}$. The frequency dependent permittivity ϵ as a function of the driving electric field with an angular frequency ω can be described by:

$$\epsilon_l(\omega) = \epsilon_l(\infty) + s_{v,l}\frac{\omega_{v,l}^2}{\omega_{v,l}^2 - i\gamma_{v,l}\omega - \omega^2}, l = x, y, z \tag{9}$$

The corresponding parameters is shown in the following Supplementary Table 1.

Moreover, in this simulation, the Au is described by Drude model, in which the local dielectric function takes the form [7]: $\epsilon_\mathrm{m} = \epsilon_\mathrm{b} - \frac{\omega_\mathrm{p}^2}{\omega^2 + i\gamma\omega}$, here $\epsilon_\mathrm{b} = 9$, $\hbar\omega_\mathrm{p} = 9$ eV, $\hbar\gamma = 0.071$ eV.

Supplementary Note 6: The reflectivity of the graphene surface with the dropped nanometer steps

As revealed by the AFM morphological comparison between the sample (left panel of Fig. 2a in the main article) and the bare Au substrate, we know that the encapsulated region is partially suspended on the holes. Therefore, the reflectivity of the graphene surface with the similar nanometer steps like the gold substrate were simulated.

By comparing the reflectance of the flat graphene and that of the dropped graphene surface, it can be known that, in such a system, whether the graphene is completely flat has a minor affect on the reflectivity of AGPs when the graphene drops less than 20%. This minor effect can be understood as following. The reflectivity depends on the impedance mismatch between acoustic graphene plasmons and graphene plasmons in the suspended region. Small variation of the suspended region leads to minor effect on the graphene

plasmons due to its strong field confinement. Therefore, assuming the graphene to be flat is a good approximation in our model.

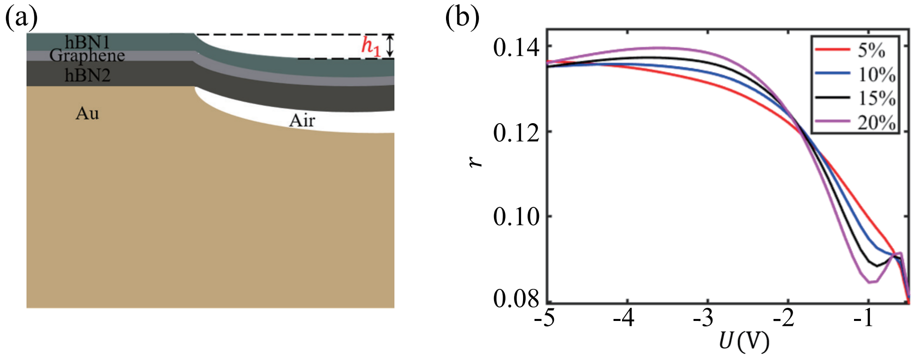

Supplementary Figure 9. The AGP reflectivity of the graphene surface with the nanometer steps. (a) The schematic diagram of the simulation model, h_1 represents the height of the graphene surface recessed downward. (b) The reflectivity with different h_1. Here, h_1=(5%, 10%, 15% and 20%) h, h is the height of the shallow steps.

Supplementary Note 7: The scattering length simulated by the three-dimensional model

In our case, the acoustic graphene plasmons incident on a nonohole instead of an interface, the scattering happens, and the near-field fringes come from the interference between the incident plasmon wave and backscattering wave. The 3D scattering model is performed here, and scattering length is obtained. The model is shown in Supplementary Figure 10(a), where the incident plasmons is scattered by a circular nanohole with the height of 3 nm and the diameter of 100 nm. The integral of the scattering energy flow is calculated. Here, the scattering energy flow is defined as $\mathbf{S} = \frac{1}{2}\text{Re}[(\mathbf{E} - \mathbf{E_0})\times(\mathbf{H} - \text{H}_0)^*]$, $\mathbf{E_0}$ and $\mathbf{H_0}$ is the electric and magnetic near-field distribution of graphene plasmons without small holes. The integration area is chosen as a cylindrical area with a radius of 0.1 μm at the bottom of the cylinder and a height of 0.26 μm. Taking into account the loss of graphene plasmons, the integrated energy flow is different in different integration ranges, therefore, the loss of graphene is ignored in the calculation. The energy flow integral is defined as P. At the same time, the energy flow without small holes is integrated on the cylindrical cross section (0.2 μm*0.26 μm) to get the incident energy flow P_0, and the final scattering length is defined

as L=P∗(0.2 μm)/P_0. And the scattering length is shown in Supplementary Figure 10(b). The normalized scattering length larger than 1 can be easily obtained for acoustic plasmons instead of traditional graphene plasmons. The valley corresponds to the position of the hBN phonon. To illustrate this point, dispersion relation of Graphene/hBN (6 nm)/Au (50 nm) and Graphene/hBN (6 nm)/SiO_2 (500 nm) were simulated and shown in Supplementary Figure 10(c) and (d). From that, it can be known the vally just falls in the forbidden zone of the dispersion relation. At the excitation wavelength of 10.653 μm (shown as the black dotted line), the scattering length of the AGPs is about 6.5 times than that of the GPs. From that it can be known the scattering ability of AGPs is much stronger than that of GPs. The result is consistent with the simplified reflection model in the main article.

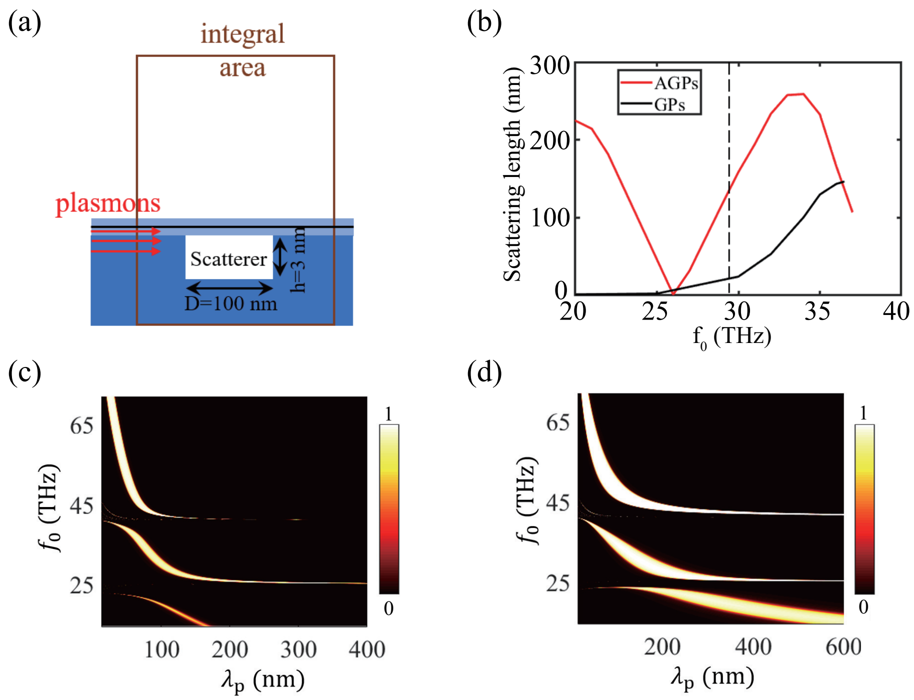

Supplementary Figure 10. The scattering length of AGPs and GPs by a nanohole with a height of 3 nm. (a) The model of calculating the energy flow integral. (b) The scattering length of AGPs and GPs. The black dotted line corresponds to 10.653 μm. (c) The dispersion relation of Graphene/hBN (6 nm)/Au (50 nm). (d) The dispersion relation of Graphene/hBN (6 nm)/SiO_2 (500 nm).

Supplementary Note 8: The AFM topography of the selected region for the second sample

Supplementary Figure 11(a) shows the AFM topography of the selected region corresponding to the black dashed square in Fig. 3a in the main article. Supplementary Figure 11(b) shows the corresponding height profile of the scatterer along the solid white line. One can find that the height of the scatterer is approximately 3 nm with the length of 100 nm.

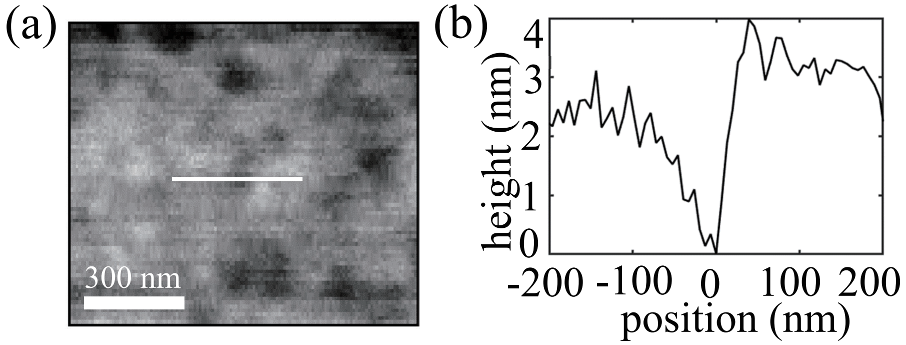

Supplementary Figure 11. (a) The AFM topography of the selected region in Fig. 3a (main article). (b) The line profile of height corresponds to the solid white line in (a).

Supplementary Note 9: Relative near-field amplitude of the encapsulated graphene boundary for the second sample

Relative near-field amplitude of the encapsulated graphene boundary (ΔS_{R}) is defined as the magnitude of the near-field amplitude of the encapsulated graphene boundary (S_{R}) relative to the near-field amplitude far from the boundary ($S_{\mathrm{R,graphene}}$). The variation of the near-field amplitude for the scatterers and the encapsulated graphene boundary is 286% and 12.47%, respectively, when the gate voltages increases from 1.6 V to 2.9 V. Therefore, ΔS_{R} can be considered almost unchanged with the change of gate voltages, which is shown in Supplementary Figure 12. The circles in the figure are the average of multiple sets of data.

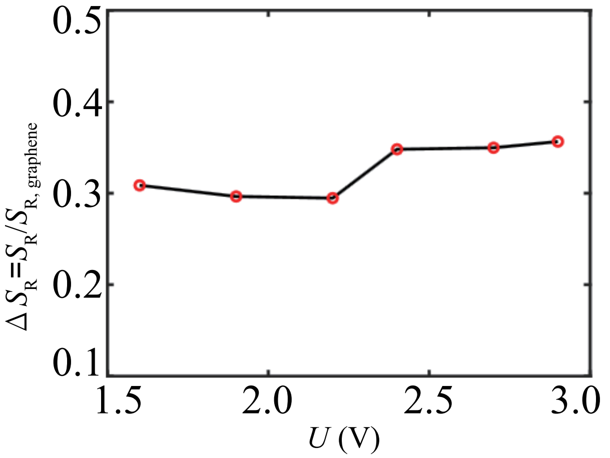

Supplementary Figure 12. Relative near-field amplitude of the encapsulated graphene boundary ($\Delta S_{\rm R}$) changes with the gate voltages. The red circles represent the data.

Supplementary Note 10: The angular distribution of the energy flow and the electric field distribution when passing the scatterers

To illustrate the scattering of AGPs by holes is more divergent in directions than that for a boundary, the angular distribution of the energy flow as passing the scatterer is shown in Supplementary Figure 13. Here, the scattering flow is defined by $\mathbf{S} = \frac{1}{2}\mathrm{Re}[(\mathbf{E}-\mathbf{E_0})\times(\mathbf{H}-\mathbf{H}_0)^*]$, $\mathbf{E_0}$ and $\mathbf{H_0}$ is the electric and magnetic near-field distribution of graphene plasmons without small holes. Supplementary Figure 13(a) and (b) show the schematic diagrams for calculating the scattered energy flow of the hole and a boundary, respectively. The intensity P of the scattered energy flow at a distance of 0.5 μm in the horizontal direction away from the center is calculated. Taking the forward direction as 0° (x direction). The calculation results show that for the hole, the scattered energy flow is mainly in the range of azimuth angle from - $\pi/4$ to $\pi/4$; while for the plane, it mainly diverges in the forward and backward directions and only within an area with a small angle. This shows that the hole scattering is indeed more divergent than plane scattering. At the same time, the electric field $|E_{\rm z}|$ distribution is simulated when propagating plasmons are scattered by a hole and a graphene/air boundary (Supplementary Figure 14). Considering that the s-SNOM signal is formed by the coherent superposition of the incident wave and the reflected wave, the electric field $|E_z|$ is selected in the backscattering direction of 0°. It can be clearly see that the electric field decays more rapidly for the point source than the

boundaries, which leads a smaller visibility for the point source.

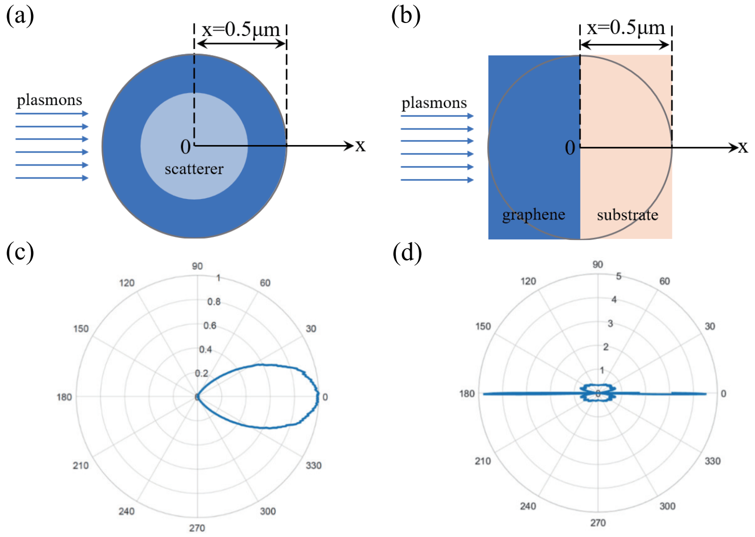

Supplementary Figure 13. The angle distribution diagram of the energy flow when passing through a single hole and the flat boundary. (a) and (b) is the schematic diagrams of calculating the scattered energy flow of a single scatterer (hole) and the graphene boundary, respectively. (c) The angular distribution of energy flow for a single scatterer. (d) The angular distribution of the energy for a boundary of graphene.

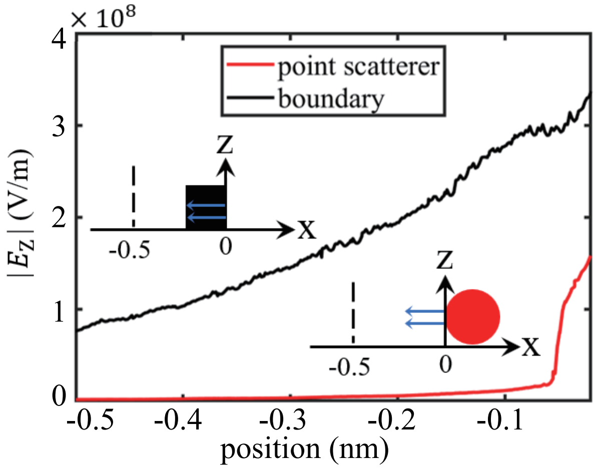

Supplementary Figure 14. The field distribution along the center position for a point scatterer and a boundary, respectively.

Supplementary Note 11: AGPs can be used as the atomic scale height sensor

Compared with traditional graphene plasmons, AGPs can be used as ultrasentive sensors due to extremely confined electric fields. For our work, one of the most obvious applications is the atomic scale height sensor, in which the height of the steps can be detected by the near-field signals. Besides, if the scattering hole is replaced with protein or chemical molecules, the ultrathin layer can be effectively recognized by AGPs. The AGPs have the potential for quantitative ultrathin protein detection and chemical-specific molecular identification. To show this potential application, the transmittance of acoustic plasmons by an atomic height protein layer is simulated. In the calculations, the analytic model is used to retrieve the protein permittivity from experimental results by adjusting a Lorentzian permittivity [8, 9]: $\epsilon_{\mathrm{p}}(\omega) = n_{\infty}^2 + \frac{S_1^2}{\omega_1^2-\omega^2-i\omega\gamma_1} + \frac{S_2^2}{\omega_2^2-\omega_2-i\omega\gamma_2}$, here, $n_{\infty}^2 = 2.08$, ω_1=1668 cm^{-1}, ω_2=1532 cm^{-1}, γ_1=78.1 cm^{-1}, γ_2=101 cm^{-1}, S_1=213 cm^{-1}, S_2=200 cm^{-1}. Supplementary Figure 15(a) and (b) shows that when AGPs and GPs pass through a nano-scale (the height of protein is 0.5 nm, 1.0 nm and 1.5 nm) protein molecule (pyridine), AGPs will form very strong transmittance peaks at the two natural frequencies of the protein molecule, but the GPs will only form absorption peaks at ω under the same circumstances. The transmittance of AGPs is significantly higher than that of GPs. It shows that AGPs have great advantages in biosensing for nano-scale protein compared to GPs.

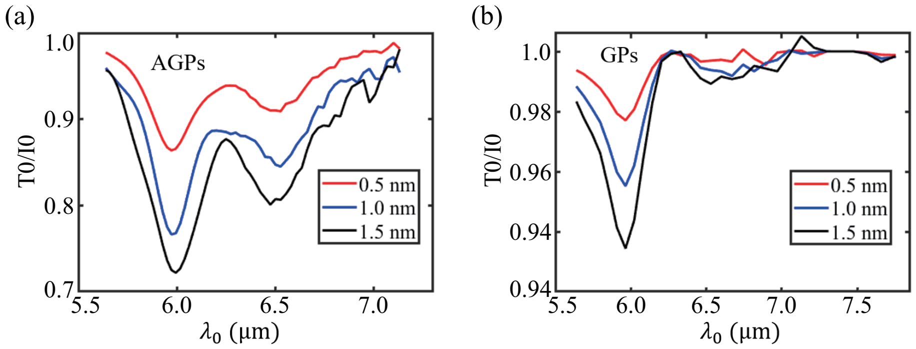

Supplementary Figure 15. The absorption spectra of the AGPs (a) and GPs (b) passing through protein molecules of different heights at 0.5, 1.0 and 1.5 nm.

Supplementary References

---

[1] Huang, Yuan et al. Reliable Exfoliation of Large-Area High-Quality Flakes of Graphene and Other Two-Dimensional Materials. *ACS Nano* **9**, 10612-10620 (2015).

[2] Zomer, P. J., Guimãres, M. H. D., Brant, J. C., Tombros, N. & Van Wees, B. J. Fast pick up technique for high quality heterostructures of bilayer graphene and hexagonal boron nitride. *Appl. Phys. Lett.* **105**, 013101 (2014).

[3] Lundeberg, Mark B. et al. Tuning quantum nonlocal effects in graphene plasmonics. *Science* **357**, 187–191 (2017).

[4] Yu, G. L. et al. Interaction phenomena in graphene seen through quantum capacitance. *Proceedings of the National Academy of Sciences* **110**, 3282-3286 (2013).

[5] Woessner, Achim et al. Highly confined low-loss plasmons in graphene–boron nitride heterostructures. *Nat. Mater.* **14**, 421–425 (2015).

[6] Paulo André D. Gonalves & Peres, Nuno M. R. The Transfer-Matrix Method and the First Appearance of Plasmons in Graphene. In *An Introduction to Graphene Plasmonics*, 35–46 (World Scientific, 2016).

[7] A. Rodríguez Echarri, Joel D. Cox & F. Javier García de Abajo Quantum effects in the acoustic plasmons of atomically thin heterostructures. *Science* **6**, 630–641 (2019).

[8] Rodrigo, Daniel et al. Mid-infrared plasmonic biosensing with graphene. *Science* **349**, 165–168 (2015).

[9] Marini, Andrea, Silveiro, Iván & García de Abajo, F. Javier Molecular Sensing with Tunable Graphene Plasmons. *ACS Photonics* **2**, 876-882 (2015).
